# The concentration of particulate matter in the barn air and its influence on the content of heavy metals in milk

**DOI:** 10.1038/s41598-023-37567-2

**Published:** 2023-06-30

**Authors:** Zenon Nieckarz, Krzysztof Pawlak, Agnieszka Baran, Jerzy Wieczorek, Jacek Grzyb, Patrycja Plata

**Affiliations:** 1 Department of Experimental Computer Physics, Institute of Physics, Jagiellonian University in Cracow, Kraków, Poland; 2 Department of Zoology and Animal Welfare, University of Agriculture in Cracow, Aleja Adama Mickiewicza 24/28, 30-059 Kraków, Poland; 3 Department of Agricultural and Environmental Chemistry, University of Agriculture in Cracow, Kraków, Poland; 4 Department of Microbiology and Biomonitoring, University of Agriculture in Cracow, Kraków, Poland

**Keywords:** Biochemistry, Chemical biology, Ecology, Zoology, Climate sciences, Ecology, Environmental sciences, Diseases, Health care, Risk factors

## Abstract

Heavy metals are one of the components of smog, which is mainly the product of burning fossil fuels in residential buildings. These elements, introduced into the body of cattle by inhalation, may enter the milk. The goal of this study was to assess the impact of particulate pollution in the atmospheric air on the concentration of particulate matter in the air of a dairy cattle barn and on the content of selected heavy metals in milk from cows present in the building. Measurements were taken between November and April (148 measurement days). The calculations carried out showed a high correlation (R_S_ = + 0.95) between the concentrations of particulates measured outside and inside the barn, which is indicative of a significant impact of the atmospheric air on the particulate pollution level of the livestock building. The number of days in excess of the daily standard for PM_10_ inside was 51. The conducted analysis of the chemical composition of the milk collected under high particulate pollution (February) showed that the permitted lead level had been exceeded—21.93 µg/kg (norm 20.00 µg/kg).

## Introduction

Health and the quality of life, both human and animal, are tightly connected to the condition of the environment. Particulate matter is one of the factors with a negative impact on the environment. The main sources of PM_10_ and PM_2.5_ are the burning of fossil fuels in electricity generation, households, transport, industry, especially steelworks, and mines^1,2^. In Europe, in large urban agglomerations where the burning of solid fuel in households has been significantly reduced, traffic remains the main source of particulate matter^3–5^. On the other hand, in rural areas, the burning of solid fuel and road traffic remain the dominant sources of particulate pollution^6^. High dust emissions, as well as adverse weather conditions (fog, lack of wind) and local topography, contribute to the development of the atmospheric phenomenon of London-type smog. It occurs most often in winter conditions, in humid air heavily contaminated with so-called acidic gases, mainly sulfur (IV) oxide (SO_2_), carbon (IV) oxide (CO_2_), nitrogen (II) oxide (NO), and dust^7^. Particles present in smog contain metal oxides, acidic condensates, heavy and transition metals^8^, sulfates and nitrates, as well as elemental and organic carbon^9^.

The harmfulness of smog is demonstrated by the fact that PM_2.5_ is considered one of the main factors that increase the risk of death^10^. The US Environmental Protection Agency^11,12^ estimates that ~ 90% of the human morbidity and mortality associated with air pollution are attributable to two primary criteria pollutants: fine particulate matter (PM_2.5_), and tropospheric ozone (O_3_). The research into the impact of smog on living organisms revealed that particle pollutants increase the risk of developing asthma and allergy^13^, cause arrhythmia^14^, lung cancer^15^ and chronic obstructive lung disease^16^, bronchitis^17^, as well as decrease fertility^18,19^ and constitute a risk factor for abnormal fetal development^20^. Recent studies also show the possibility of a link between the high level of mortality caused by the severe acute respiratory syndrome CoronaVirus 2 and particulate contamination of the atmosphere^21,22^. Data that clearly demonstrates the harmfulness of particulates makes one issue more stringent standards and recommendations regarding the concentration of these particles in the air. According to the new guidelines provided by WHO^23^, the maximum daily concentration of PM_2.5_ should not exceed 15 µg/m^3^ (previously 25 µg/m^3^), and for PM_10_ the maximum permitted concentration should not be greater than 45 µg/m^3^ (previously 50 µg/m^3^). It should be noted that there is no standard in the European Union legislation for the daily concentration of PM_2.5_, while the daily standard^24^ for PM_10_ is 50 µg/m^3^.

Studies by Massey et al.^25^ as well as Chen et al.^26^ showed that, in the event of high atmospheric pollution, particles of particulate matter may enter houses. This problem is particularly important in animal buildings where dust reaches not only through window openings and leaks but also through supply ventilation, which is a permanent and essential part of proper equipment in animal facilities^27^.

As mentioned above, heavy metals may be one of the components of particulate matter. Heavy metals present in particulates through the respiratory system enter the blood from where the blood system distributes them throughout the body. Studies have shown that despite the fact that the mammary gland forms a natural biological barrier reducing the passage of toxic elements from the cow to milk, at high concentrations heavy metals may enter the product^28^. As it is known, milk and its preserves are one of the main sources of animal protein in the daily human diet. Therefore, research into heavy metals and their sources of origin is particularly important.

Earlier research by the authors of this article on dustiness in the building of the antelope house at the Silesian Zoological Garden^27^ showed a significant impact of smog on the dust content in rooms for these animals. The obtained results prompted the authors to undertake further research on the impact of pollution on animals and products of animal origin. The goal of this study is to assess the impact of particulate pollution in the atmospheric air on the concentration of particulate matter PM_10_ and PM_2.5_ in the air of a dairy cattle barn and on the content of selected heavy metals in milk from cows present in the building.

## Materials and methods

### Ethics statement

In our study, we did not expose animals to artificially created factors that were unfavorable to them, but we only observed the conditions in a commercial dairy cattle shed. We have not made any changes and we have not introduced any new factors into the barn. Throughout our study described in the article, none of the researchers had contact with cows. Our research was only focused on automatic registration of dust concentration and laboratory (outside the barn) assessment of the composition of milk and food. The milk used for the research was a part of the product intended for sale, coming from standard milking performed by employees employed in the barn, also without the participation of researchers.

Such activities, in accordance with the Directive of DIRECTIVE 2010/63/EU OF THE EUROPEAN PARLIAMENT AND OF THE COUNCIL of 22 September 2010^29^ on the protection of animals used for scientific purposes, do not require the consent of the ethics committee.

### Research object

**Figure 1:**
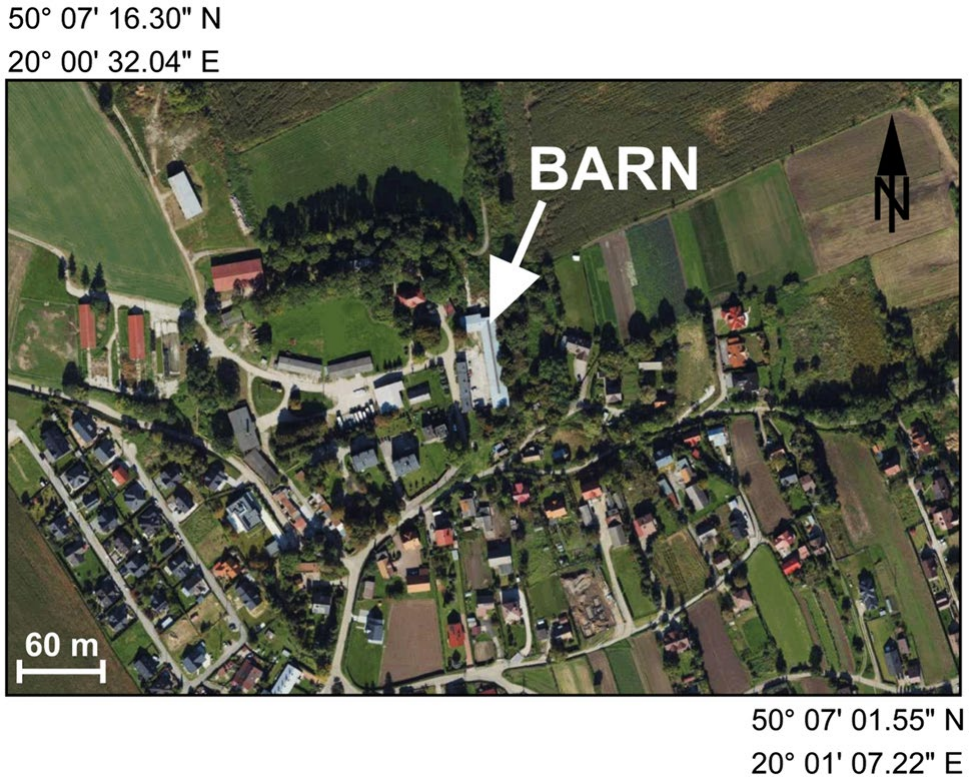
A fragment of an orthophotomap showing the location of the area where the measurements were taken (based on the website http://www.geoportal.gov.pl. The final version of the map was created using—OriginPro 2023b https://www.originlab.com/Newst).

The study was carried out between 14 November 2019 and 09 April 2020 (148 days), at a time when the lowest temperatures during the winter of 2019/2020 were recorded. Measurements of particulate matter concentration were carried out in a dairy cattle barn in the village of Dziekanowice (820 residents). The barn is situated in the vicinity of residential buildings (Fig. 1). Cows on this farm are kept in the barn for the entire year.

**Figure 2:**
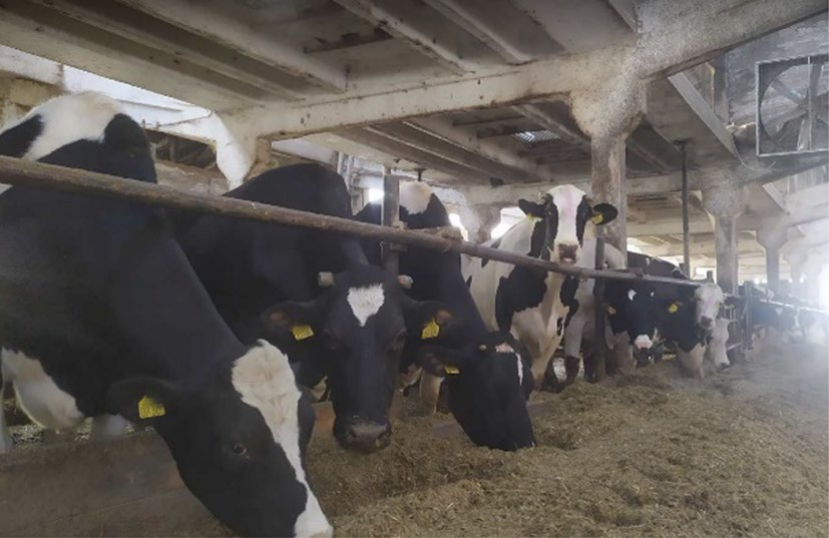
Fragment of the examined barn with cows.

In the house, there were 84 dairy cows of the Holstein–Friesian breed with an average weight of 560 kg. The barn has no ceiling, it has full walls and 20 windows (10 on each long wall) (Fig. 2). Building dimensions: width—10.39 m, length—54.87 m, height at the lowest point—3.82 m, height at the highest point—5.37 m, cubature—2618 m^3^. The animals were kept tethered in the alcove system, on a concrete floor covered with litter (long wheat straw with a moisture content of approximately 8%). The animals were fed the same TMR feed throughout the period of the experiment. The feed was fed using an electrically powered feed-supplying robot. The water used by the animals was supplied by the water distribution system of the Krakow Water company. This water is constantly tested for metal content as well. During the experiment, no exceedances of the standards for the quality of water used by animals were observed. The manure was removed manually. Cleaning and litter replacement were done once daily in the morning between 7:00 and 9:00. The building was not heated. Standard natural gravitational/ridgepole ventilation (exhaust vents are located at the top of the roof, in the ridgepole, supply vents are above the windows) and air mixers were used in the barn. The ventilation capacity was 9400 m^3^/h. The ventilation did not have any air filters. During the measurement period, the air mixers were not used. The ventilation operates 24/7. The ventilation system was not cleaned during taking measurements.

### Methods of measurement

During the tests, measurements of air temperature, relative air humidity, and PM_10_ and PM_2.5_ particulate concentrations were carried out. Measurements were performed using university measuring stations (UMS) developed as part of the Storm&DustNet scientific project implemented at the Jagiellonian University in Krakow^30^. This device (UMS) is equipped with, among other things, laser sensor SEN0177, which allows to measure PM_10_ and PM_2.5_ concentrations in the air. One measuring device is installed in the center of the tested object, 2 m above the floor, and the second one outside, 2 m above the ground at a distance of 28 m from the tested object. The particulate matter concentration and other air parameters were recorded continuously, with the mean values being recorded every minute. The accuracy of the particulate matter sensor had been verified in the previous calibration and test measurements using the reference analyzer EDM107 produced by GRIMM (GRIMM Aerosol Technik Ainring GmbH & Co. KG, Germany). The measurement error of the EDM107 analyzer is ± 2 μg/m^3^, which is confirmed by a calibration certificate and equivalence to the gravimetric method^31^. The performed calibration measurements showed that the results of measurements of PM_2.5_ and PM_10_ in UMS stations are affected by an error not exceeding ± 9 μg/m^3^. The temperature and humidity measurements were made using the BME280 sensor integrated into the UMS stations (temperature—measuring range: − 40 to + 85 °C, accuracy: ± 1 °C; humidity: measuring range: 10–100% RH; accuracy: ± 3% RH).

### Determination of selected heavy metals in milk and feed samples

We chose metals which are deemed highly hazardous and may be present in dust particles for the determination in milk and feed cadmium (Cd), chrome (Cr), copper (Cu), nickel (Ni), lead (Pb), zinc (Zn).

### Milk

Milk and feed samples were taken twice, on 15 February 2020 (period of high smog concentration) and 09 April 2020 at the end of the experiment (period of low smog concentration). The milk was collected by individual hand milking of 100 ml from the same 7 cows. After transfer to the laboratory, 50 ml was pipetted from each sample into quartz evaporators in two replicates. After adding 1 ml of concentrated HNO_3_, the contents were evaporated to dry on a hot plate. The samples were then digested in a muffle furnace at 450 °C for 4 h. The residue after burning was treated with 8 ml of the mixture of HNO_3_ and HClO_4_ acids (3:1). Evaporating dishes were covered with watch glasses and heated on a hot plate for 1 h, and then the acids were evaporated to dryness. Afterwards, 5 ml of HNO_3_ (1:2) was added, covered with a watch glass and heated for 1 h. The samples were filtered into 25 ml graduated flasks. The element content in the filtrates was determined using the inductively coupled plasma atomic emission spectrophotometer (ICP-OES) from PerkinElmer, model Optima 7300 DV.

### Feed

The feed was collected from 7 randomly selected points from the feed robot just before giving it to the cows. 100 g of feed was taken from each point. The feed samples were dried at 60 °C and then ground in a laboratory mill. The ground and homogenized material was weighed 4 g from each collected sample into quartz evaporating dishes, in two replicates. The samples were burned in a muffle furnace at 450 °C for 12 h. After removal and cooling, the ash was treated with 5 ml of HNO_3_ solution (1:2), evaporated to dryness on a hot plate, and then burned in a furnace at 450 °C for 3 h. After removal from the oven, the samples were treated with 5 ml of HCl (20%), evaporated to dryness, treated with 5 ml of HNO_3_ (1:2), covered with a watch glass and digested on a hot plate for 1 h, and then filtered to 25 ml volumetric flasks. The element content in the prepared solutions was determined using the inductively coupled plasma atomic emission spectrophotometer (ICP-OES) from PerkinElmer, model Optima 7300 DV.

### Statistical analyzes

Statistical analyzes were carried out using OriginPro 2016 (OriginLab Corporation, Northampton, MA, USA). A test on the normality of distributions of the observed variables was performed by the Shapiro–Wilk test. Since the collected measurement results do not have a normal distribution, they were analyzed using the Spearman correlation coefficient (R_S_), together with determining the significance level of the p-value. In all cases, a 2-tailed test of significance was used. We also used OriginPro to fit the linear model to the data.

## Results

**Figure 3:**
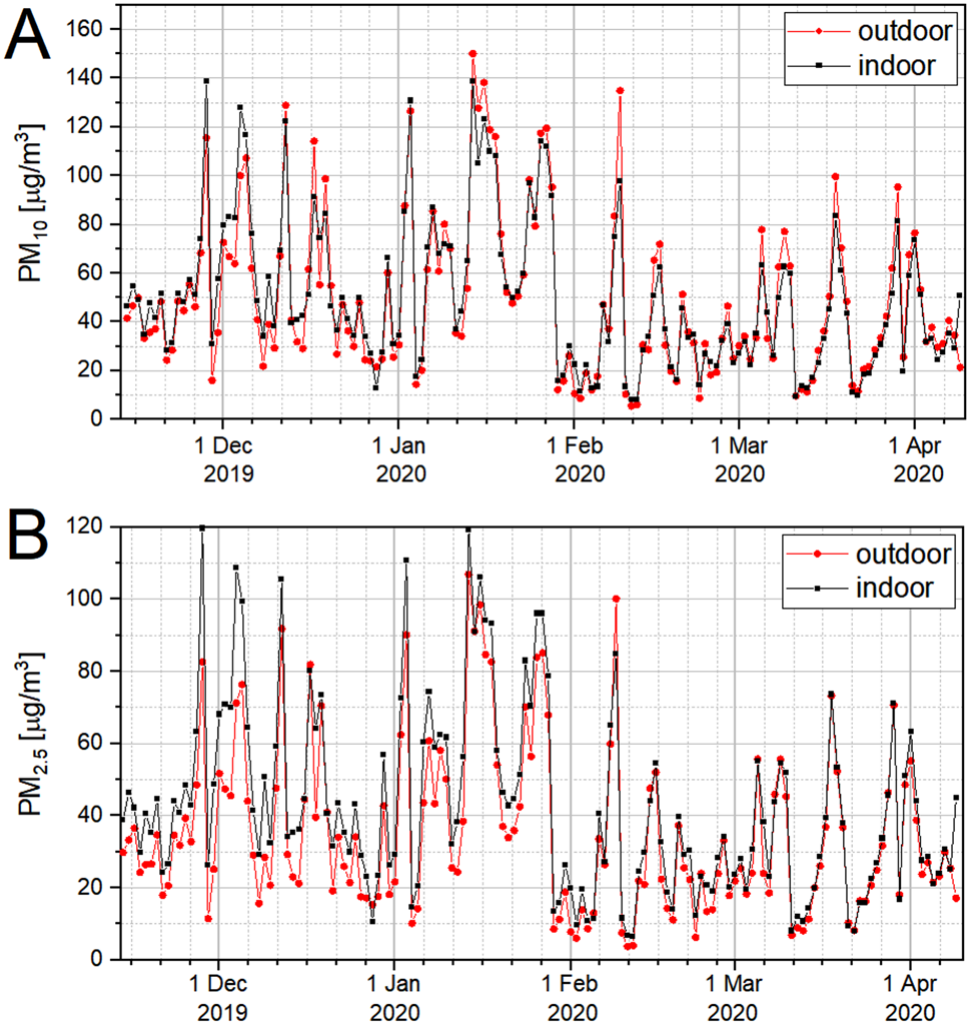
Distribution of daily average PM_10_ concentration (**A**) and PM_2.5_ concentration (**B**) determined based on measurements taken inside (black) and outside (red) the barn in the period from 14 Nov 2019 to 09 Apr 2020.

Based on the conducted measurements of particulate concentrations, daily averages for PM_10_ and PM_2.5_ were determined. The course of the daily average these dusts concentrations for studied period is shown in Fig. 3.

Outdoor measurements showed 53 days when the level of 50 µg/m^3^ (EU standard) was exceeded, and when new WHO guidelines on the concentration of these particulates (45 µg/m^3^) were taken into account, the number of such days was 67. The highest values of PM_10_ concentration was recorded on 14 January 2020—150.0 µg/m^3^. According to the measurements carried out inside the barn, there were 51 days of excessive particulate concentration (taking into account the new WHO recommendations—70 days). The highest average daily concentration for PM_10_ inside the building were observed on 14 January 2020 and 28 November 2019—138.8 µg/m^3^.

The Spearman correlation coefficient (R_S_), calculated for the whole studied period, showed a strong (p < 0.01) relationship between PM_10_ concentrations outside and inside the building (R_S_ = + 0.95), and the slope of the linear fit was 1.03 ± 0.03 (p < 0.001).

The course of the daily average PM_2.5_ concentrations for the studied period is shown in Fig. 3. Over the studied period, compared to the old WHO guidelines, there were 79 days with the exceeded level of 25 μg/m^3^ outside the building and 107 days inside. When taking into account the new WHO proposals regarding the daily PM_2.5_ standard for outdoors, there were 121 days with exceeded levels outside the barn and 128 days inside it. The highest average-daily PM_2.5_ concentration outside the houses was recorded on 14 January 2020—106.9 μg/m^3^, and inside the barn on 28 November 2019—119.7 μg/m^3^.

The conducted statistical tests showed a strongly (R_S_ = + 0.95) significant correlation (p < 0.01) between the PM_2.5_ concentration in atmospheric air and inside the barn, and the slope of the linear fit was 0.86 ± 0.02 (p < 0.001).

**Figure 4:**
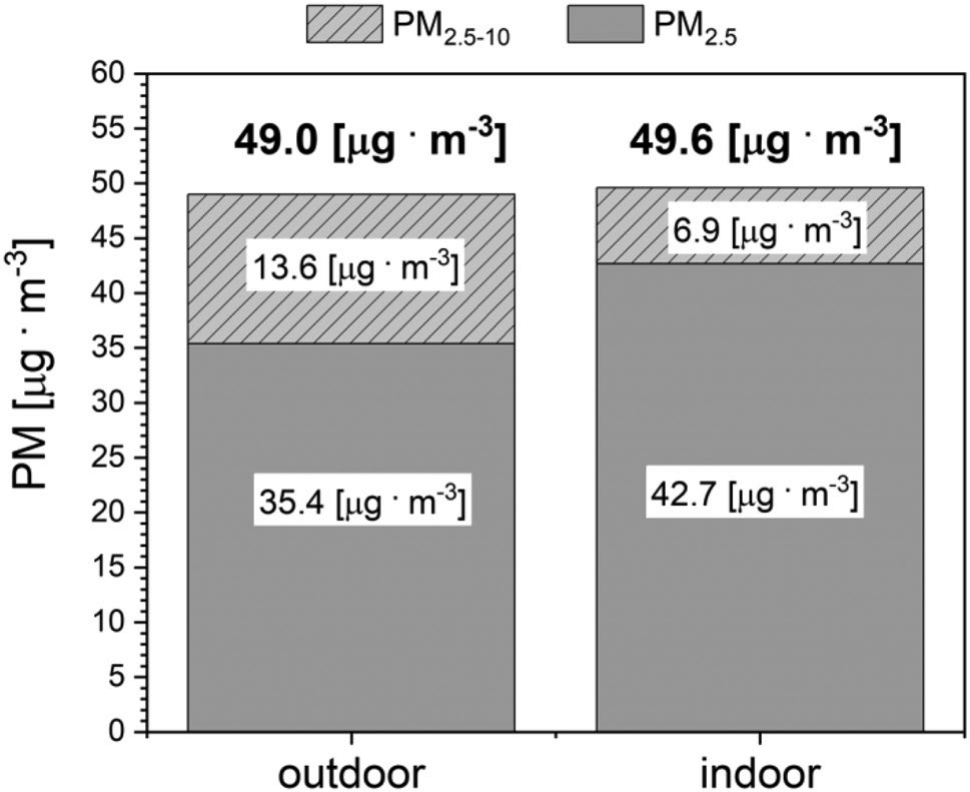
PM_10_ concentrations outside and inside the barn, divided into PM_2.5_ and PM_2.5–10_ fractions.

The statistical analysis carried out showed a very strong correlation between the PM_10_ and P_2.5_ concentration levels in both outdoor and indoor air. The Spearman correlation coefficient between PM_10_ and PM_2.5_, both inside and outside, was + 0.99 (p < 0.001). The computed average concentrations of PM_10_ outside and inside the barn between 14 November 2019 and 09 April 2020 were almost identical (49.6 μg/m^3^ inside the building, 49.0 μg/m^3^ outside) (Fig. 4).

**Figure 5:**
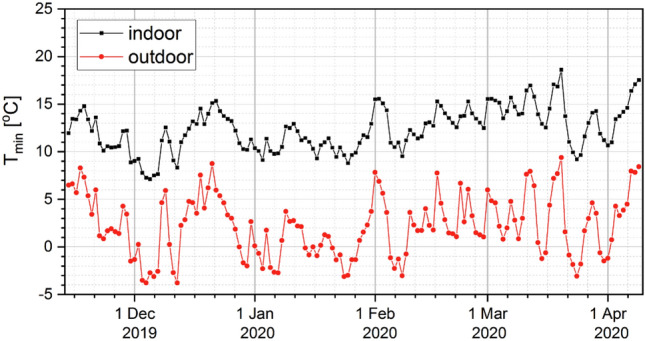
The course of daily minimum temperatures determined from the measurements taken inside (black) and outside (red) the barn in the period from 14 Nov 2019 to 09 Apr 2020.

The course of minimum daily temperatures over the studied period is shown in Fig. 5. Daily minimum temperatures outside the building ranged from − 3.7 to 9.4 °C, and inside the barn, they reached values between 7.1 and 18.6 °C. In the course of the tests, there were 42 days with sub-zero temperatures outside. The statistical calculations performed showed a clear negative relationship between the daily outdoor temperature and the particulate concentration inside the studied building (for PM_2.5_: R_S_ = − 0.32; for PM_10_: R_S_ = − 0.33).

**Figure 6:**
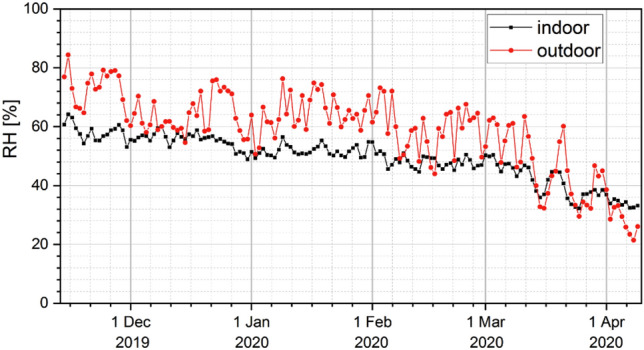
The course of average daily values of relative humidity, determined from the measurements taken inside (black) and outside (red) the barn in the period from 14 Nov 2019 to 09 Apr 2020.

The relative air humidity, measured outside the building, was between 27 and 84%, while the indoor humidity was between 32 and 64% (Fig. 6). The calculated correlation coefficient between relative humidity of the atmospheric air and particulate concentration (both PM_10_ and PM_2.5_) was R_S_ = + 0.18.

The average concentrations of heavy metals determined in fresh milk are presented in Table 1.

**Table 1:** Mean values of heavy metals determined in fresh milk taken from cows present in the barn where particulate pollution measurements were carried out. *Statistically significant difference within the column at p ˂ 0.05; **statistically significant difference within the column at p ˂ 0.01.

Collection date	Cd (µg/kg)	Cr (µg/kg)	Cu (µg/kg)	Ni (µg/kg)	Pb (µg/kg)	Zn (µg/kg)
15 Feb 2020 Period of high particulate concentration	0.28**	16.09	168.92	4.99	21.93**	5280.40
09 Apr 2020 Period of low particulate concentration	0.07**	14.93	153.81	4.07	14.14**	5260.10

The analysis carried out showed statistically significant differences in the cadmium and lead content between milk samples taken during high smog concentrations (15 Feb 2019) and those collected at low concentrations of particulate matter in the air (09 Apr 2020). The determined heavy metal content of the feed is shown in Table 2. The samples taken from the feed fed to the animals did not show any differences between the samples taken in February and April.

**Table 2:** Mean values of the heavy metals indicated in the feed used to feed the cows from which the milk was collected.

Collection date	Cd (mg/kg)	Cr (mg/kg)	Cu (mg/kg)	Ni (mg/kg)	Pb (mg/kg)	Zn (mg/kg)
15 Feb 2020 Period of high particulate concentration	0.15	12.66	13.34	8.05	1.51	92.80
08 Apr 2020 Period of low particulate concentration	0.16	12.13	17.44	8.51	0.98	79.41

## Discussion and conclusions

The measurements carried out showed that in the studied area, high concentrations of particulate matter are present in atmospheric air, often exceeding the existing standards and recommendations for PM_10_ and PM_2.5_. The high level of particulate pollution found by the authors of this article is confirmed by reports assessing the air quality in the Małopolska province where the study was conducted^32,33^. The area surrounding the cowshed building, which is situated in rural areas (Fig. 1), lacks any substantial industrial facilities, power plants, or roads with heavy traffic. The burning of fossil fuels appears to be the main source of particle air pollution^6^. The observed high concentration of particulates in atmospheric air translated into high particulate pollution in the studied building, as shown by the high values of the slopes of the linear fit and the high correlation between the mean daily concentrations of PM_10_ and PM_2.5_, both outside and inside the tested house. It must have been influenced by the gravity ventilation in that house, the ridgepole, which introduced air from the outside into the building without any filtration system. Pawlak and Nieckarz^27^ as well as Wenke et al.^34^ also pointed out the significant effect of high concentrations of particulates in atmospheric air on the air quality inside animal houses. Similar results, but regarding the effects of smog on the amount of particulate matter in human houses, were presented by Challoner and Gill^35^ as well as Massey et al.^25^.

The number of particulate particles transported with air to the respiratory system depends, among other things, on the volume of air inhaled by animals, i.e. on the respiratory volume of the lungs, reduced by the so-called dead breathing space. For resting dairy cattle, the volume of air inhaled is approximately^36^ 9 dm^3^. Assuming that the average number of breaths per minute for these animals at rest is 30, it can be estimated that during the period of the highest particulate pollution recorded (PM_10_ = 138 μg/m^3^, PM_2.5_ = 119 μg/m^3^), cows introduced approximately 2235 μg of PM_10_ into the respiratory system within an hour, including 1928 μg of PM_2.5_.

The high resting oxygen demand, low lung efficiency, increased respiratory rate during the mating season, and high metabolic requirements make dairy cows particularly vulnerable to the adverse effect of excessive particulate pollution^37,38^.

Heavy metals are one of the documented components of particulate matter^8,39^. Heavy metals entering a cow’s body through the alimentary or respiratory tract may enter the milk^40^. The analysis of the chemical composition of the milk collected during the above-mentioned experiment showed statistically significant differences between the content of cadmium and lead in the samples of this raw material taken during the period of high and low particulate air pollution. In the case of other heavy metals determined in the milk, no such differences were found. The conducted determination of the feed composition as well as information on the quality of water drunk by animals exclude the impact of these factors on the increase in lead and cadmium content in the milk that had been collected during a period of high particulate pollution^41,42^.

Both Cd and Pb are elements that do not have any biological role in the animal’s body, but cause toxic effects even at very low concentrations^43,44^. When comparing the average concentrations of lead in the studied milk with the permissible level of Pb (0.020 mg/kg fresh matter)^45,46^, it was established that the content of this metal in the studied raw material was exceeded during the period of high particulate pollution. In the available literature, we can find reports of excessive lead content in cow milk from animals maintained in industrial areas^47,48^, near motorways^49^ or municipal facilities^50^, while there are no studies discussing the impact of smog on the presence of Pb in the milk of cows kept in barns.

At present, there are no precise standards for acceptable cadmium levels in milk. The maximum permitted level for Cd in milk proposed in different countries^51^ is between 2.0 and 10 μg/kg. The recommended level of cadmium in the studied milk was exceeded neither during high nor low particulate pollution. Since cadmium is one of the most toxic heavy metals, studies on its content in milk are often carried out^48,50,52^. However, similarly to lead, there are no studies regarding the effect of smog on the presence of Cd in cow milk from animals kept in barns.

In the climatic conditions prevailing in central Europe, dairy cattle are kept in animal houses for almost the entire autumn and winter period. A large proportion of these buildings are equipped with filter-free gravitational ventilation^53,54^. Consequently, in areas with high air pollution, a large proportion of cattle may be exposed to excessive particulate pollution, which will adversely affect their health and the quality of animal-based products. This is particularly dangerous in the case of milk, as this product is one of the main sources of protein in the human diet (111.6 thousand tons of milk products was produced in 2020 in the European Union^55^). The observed global increase in coal and gas prices and the emerging shortages of these raw materials make the increase in the use of lower-quality fuels used for heating houses highly likely. This will lead to an increase in particulate pollution, thereby deteriorating the air quality in animal buildings. In view of the above, it should be concluded that further studies are necessary on the impact of smog on the concentration of particulate matter in the air of dairy cattle barns and on the content of heavy metals in milk. It seems that research on the impact of smog on the presence of harmful substances in other products of animal origin should also be carried out.

## Data Availability

All data generated or analyzed during this study are included in this published article. All materials are housed on Department of Experimental Computer Physics, Institute of Physics, Jagiellonian University Cracow, Poland. The datasets used and/or analysed during the current study available from the corresponding author on every request.
